# Uterine Nodal expression supports maternal immunotolerance and establishment of the FOXP3^+^ regulatory T cell population during the preimplantation period

**DOI:** 10.3389/fimmu.2023.1276979

**Published:** 2023-10-30

**Authors:** Sarah Yull, Shiva Shafiei, Craig B. Park, Parinaz Kazemi, Emily B. Tiemann, Marie-Hélène Godin Pagé, Daniel Dufort

**Affiliations:** ^1^ Division of Experimental Medicine, McGill University, Montreal, QC, Canada; ^2^ Child Health and Human Development Program, Research Institute of the McGill University Health Centre, Montreal, QC, Canada; ^3^ Department of Obstetrics and Gynecology, McGill University, Montreal, QC, Canada; ^4^ Department of Biology, McGill University, Montreal, QC, Canada

**Keywords:** Nodal, pregnancy, female infertility, recurrent implantation failure, preimplantation period, inflammation, maternal tolerance, regulatory T cells

## Abstract

Pregnancy success is dependent on the establishment of maternal tolerance during the preimplantation period. The immunosuppressive function of regulatory T cells is critical to limit inflammation arising from implantation of the semi-allogeneic blastocyst. Insufficient maternal immune adaptations to pregnancy have been frequently associated with cases of female infertility and recurrent implantation failure. The role of Nodal, a secreted morphogen of the TGFβ superfamily, was recently implicated during murine pregnancy as its conditional deletion (Nodal^Δ/Δ^) in the female reproductive tract resulted in severe subfertility. Here, it was determined that despite normal preimplantation processes and healthy, viable embryos, Nodal^Δ/Δ^ females had a 50% implantation failure rate compared to Nodal^loxP/loxP^ controls. Prior to implantation, the expression of inflammatory cytokines MCP-1, G-CSF, IFN-*γ* and IL-10 was dysregulated in the Nodal^Δ/Δ^ uterus. Further analysis of the preimplantation leukocyte populations in Nodal^Δ/Δ^ uteri showed an overabundance of infiltrating, pro-inflammatory CD11b^high^ Ly6C^+^ macrophages coupled with the absence of CD4^+^ FOXP3^+^ regulatory T cells. Therefore, it is proposed that uterine Nodal expression during the preimplantation period has a novel role in the establishment of maternal immunotolerance, and its dysregulation should be considered as a potential contributor to cases of female infertility and recurrent implantation failure.

## Introduction

Female infertility is defined as the inability to establish or maintain pregnancy and affects approximately 15% of women of reproductive age. As the age women attempt to conceive their first child steadily increases, consequences of advanced maternal age including a higher incidence of infertility and a greater reliance on assisted reproductive technologies (ART) have become unavoidable (1). Infertility can arise from defects at any of the critical events during early reproduction such as irregular or failed ovulation, tubal obstruction, reproductive tract dysfunction or pathological conditions like endometriosis (2, 3). However, unexplained infertility still accounts for a substantial portion of cases (2). Although several forms of female infertility can be overcome with *in vitro* fertilization, recurrent implantation failure is not easily circumvented with ART (4). Importantly, these underlying reproductive conditions as well as the use of ART pose a significant risk for later pregnancy complications like preeclampsia or preterm labor (5–7). Therefore, elucidating mechanisms that contribute to reproductive pathologies and infertility is fundamental for the improvement of maternal and fetal health outcomes.

Pregnancy is established once the embryo implants into the uterine wall after oocyte fertilization and transport through the fallopian tube (oviduct). The uterine lumen and endometrium are conditioned by ovarian steroid hormones into a receptive and competent state required for embryo implantation. Precisely orchestrated and reciprocal signaling between the receptive uterus and the free-floating blastocyst mediates the apposition, attachment and invasion of the embryo into the uterine endometrium. Numerous factors such as cytokines, growth factors and morphogens have been implicated in the molecular crosstalk of implantation but the precise role of many of these components remains undefined (8–12).

An integral concept of pregnancy is the dynamics of the maternal immune system in response to the semi-allogeneic fetus. Implantation and the early events of placentation are considered pro-inflammatory, as the breakdown of the uterine epithelium, invasion of the blastocyst and vasculature remodeling is mediated by the infiltration and activation of leukocytes to assist in endometrial repair. This local inflammation in the uterus is controlled by regulatory T cells (Tregs) which have anti-inflammatory and immunosuppressive functions to support maternal tolerance while preventing the rejection of the fetus. Balance between these two states is necessary for successful implantation and pregnancy maintenance, therefore any dysregulation or challenge from external inflammation could risk the viability of pregnancy (13–17). Indeed, reproductive pathologies that present across all stages of gestation have been extensively correlated with inadequate maternal immune adaptations to pregnancy. For cases of unexplained infertility and specifically recurrent implantation failure Tregs are a commonly implicated population (18, 19).

The expression, regulation and function of Nodal, a morphogen of the TGFβ superfamily, was previously described during murine pregnancy. Nodal is expressed throughout the uterine glandular epithelium during the preimplantation period following mating. At the time of embryo apposition and attachment, Nodal is expressed exclusively in the areas between pre-emptive implantation sites and is embryo-dependent, implying a critical function for Nodal during the crosstalk of implantation (20). The contribution of TGFβ and other superfamily members to the molecular and immunomodulatory events of pregnancy has been well characterized (19, 21, 22), but the specific function of uterine Nodal in facilitating successful reproduction remains unknown. Previously, the generation of a maternal reproductive tract-specific Nodal knockout mouse strain (Nodal^Δ/Δ^) demonstrated multiple reproductive phenotypes including a reduced pregnancy rate at term, smaller litter size and pups with intrauterine growth restriction (23). Interestingly, at later stages of pregnancy heterozygous Nodal^Δ/+^ uteri had premature elevation of the pro-inflammatory cytokines IL-1β, IL-6 and TNF-α, and increased infiltration of decidual macrophages. This premature pro-inflammatory response during the expected stage of sustained anti-inflammatory tolerance caused a greater susceptibility for LPS-induced preterm birth, and it was proposed that uterine Nodal expression supported an anti-inflammatory state during the later stages of pregnancy (24).

Despite significant subfertility in Nodal^Δ/Δ^ females the role of Nodal during early reproduction has not been considered. Therefore, the focus of this study is implantation and the establishment of pregnancy using the Nodal^Δ/Δ^ model. Here, a novel role for uterine Nodal expression in supporting maternal immunotolerance during the preimplantation period is proposed. Nodal^Δ/Δ^ females experience implantation failure which is largely attributed to the lack of a CD4^+^ FOXP3^+^ Treg population in the uterus prior to implantation.

## Results

### Nodal deficient females are subfertile and have implantation failure

**Figure 1:**
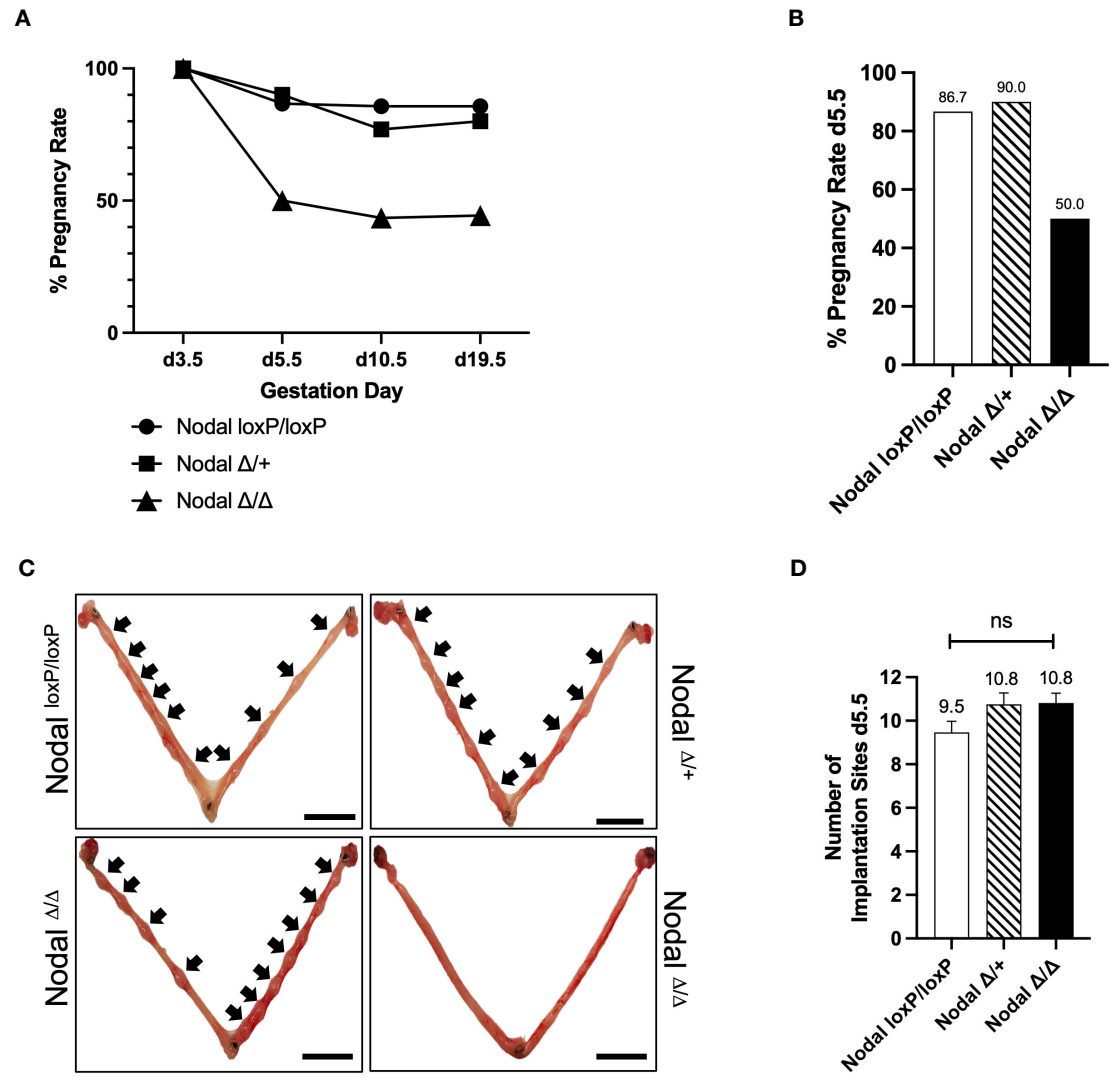
Nodal^Δ/Δ^ females have implantation failure. **(A, B)** The pregnancy rate of Nodal^loxP/loxP^, Nodal^Δ/+^ and Nodal^Δ/Δ^ females during independent dissection experiments across multiple gestation days. Nodal^Δ/Δ^ mice have a significant reduction in pregnancy rate between d3.5 and d5.5 (d3.5 Nodal^loxP/loxP^ n=6, Nodal^Δ/+^ n=2, Nodal^Δ/Δ^ n=9, confirmed by the presence of embryos after flushing; d5.5 Nodal^loxP/loxP^ 87.6% (n=15), Nodal^Δ/+^ 90.0% (n=10), Nodal^Δ/Δ^ 50.0% (n=22); d10.5 Nodal^loxP/loxP^ 85.7% (n=7), Nodal^Δ/+^ 76.9% (n=13), Nodal^Δ/Δ^ 43.5% (n=23), d19.5 Nodal^loxP/loxP^ 85.7% (n=7), Nodal^Δ/+^ 80.0% (n=5), Nodal^Δ/Δ^ 44.4% (n=9)). **(C)** Representative whole mount d5.5 uteri show implantation sites (black arrows) of pregnant Nodal^loxP/loxP^, Nodal^Δ/+^ and Nodal^Δ/Δ^ females. Nodal^Δ/Δ^ mice that were plugged but not pregnant have no implantation sites on d5.5, demonstrating complete implantation failure. Scale bars indicate 1 cm. **(D)** Nodal^Δ/Δ^ females that had visible implantation sites on d5.5 had a similar number of sites compared to Nodal^loxP/loxP^ controls (Nodal^loxP/loxP^ n=13, Nodal^Δ/+^ n=8, Nodal^Δ/Δ^ n=11). Data shows mean ± SEM.

The generation of a reproductive-tract specific deletion of Nodal using the *Pgr*-Cre and Nodal^loxP/loxP^ strains was previously described (23). Nodal deficiency in both Nodal^Δ/+^ and Nodal^Δ/Δ^ female mice resulted in significant subfertility, which was more drastic in Nodal^Δ/Δ^ females (23, 24). In order to understand the pathophysiology of this reduced fertility, the pregnancy status of Nodal^loxP/loxP^ controls, Nodal^Δ/+^ heterozygotes and Nodal^Δ/Δ^ knockout females was determined across multiple stages of gestation. Mice were mated overnight with wild-type CD1 males and the presence of a copulatory plug the following morning indicated day 0.5 post coitum (d0.5). The pregnancy rate was evaluated by independent dissection experiments on d3.5 (confirmed by the presence of embryos), d5.5 and d10.5, or the birth of a litter at term on d19.5 (**Figure 1A**). On d3.5, flushed uteri of all females contained embryos with a normal morphology. Following implantation, Nodal^loxP/loxP^ and Nodal^Δ/+^ mice showed a similar pregnancy rate ranging between 80-90% at each gestational stage assessed. However, the pregnancy rate of Nodal^Δ/Δ^ females was considerably decreased to 50% on d5.5 (**Figure 1B**). Whole mount uteri from plugged and pregnant d5.5 Nodal^loxP/loxP^ and Nodal^Δ/+^ females showed implantation sites as visible swellings along each uterine horn (**Figure 1C**, black arrows). Surprisingly, among the 50% of Nodal^Δ/Δ^ females that could progress past implantation there was no difference in the number of implantation sites seen on d5.5 (Nodal^loxP/loxP^ 9.5 ± 0.5, Nodal^Δ/+^ 10.8 ± 0.5, Nodal^Δ/Δ^ 10.8 ± 0.4 sites/female) (**Figure 1D**). Although pregnant Nodal^Δ/Δ^ females too had numerous, visible implantation sites, the total absence of sites in 50% of plugged mice suggested “all or nothing” implantation.

**Figure 2:**
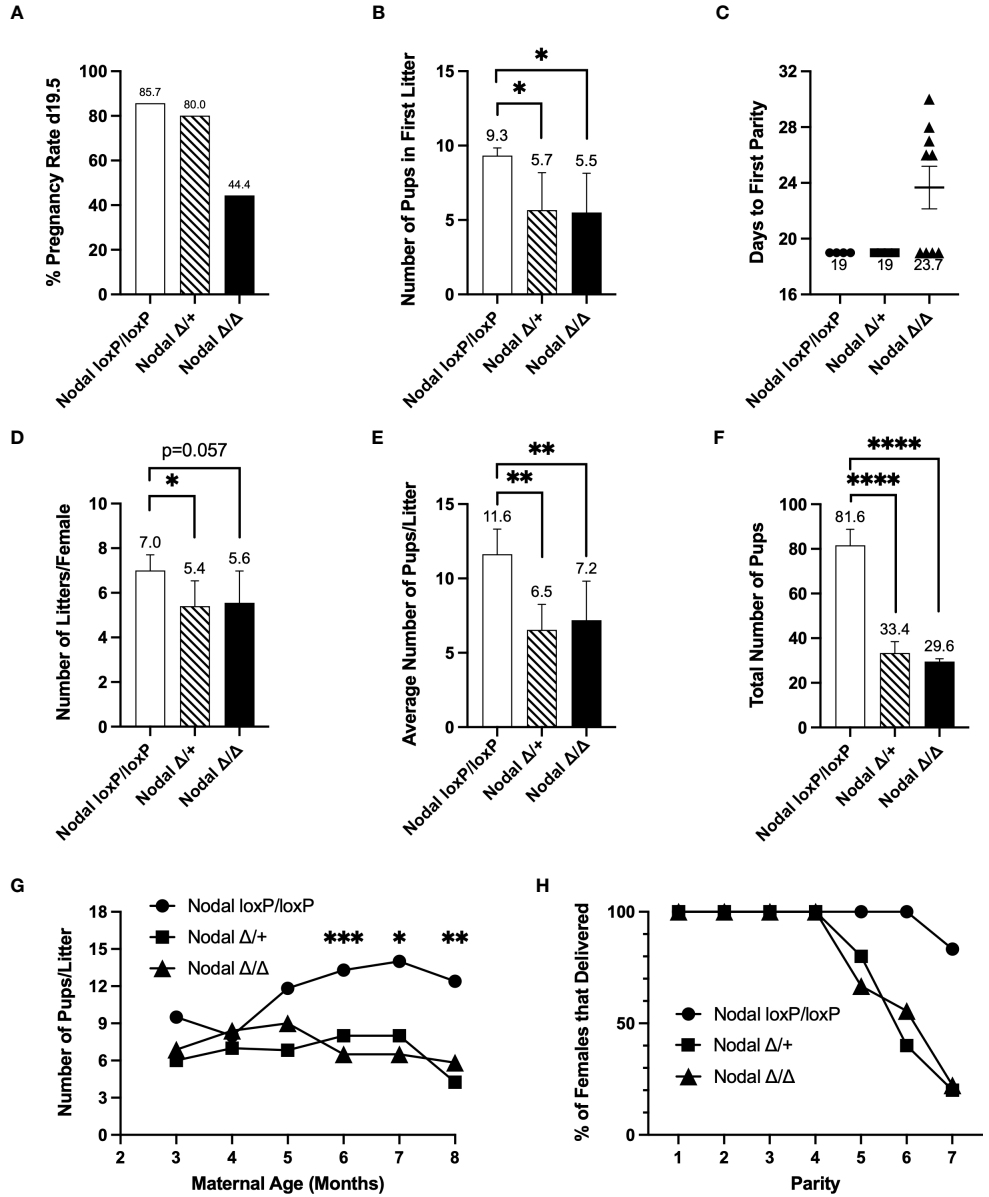
Nodal-deficient females are subfertile. Eight-week-old Nodal females were mated and housed with wildtype CD1 males to be observed during a six-month breeding period. After confirmation of the first copulatory plug (d0.5), **(A)** the initial pregnancy rate of Nodal^Δ/Δ^ mice, defined as the percentage of females that delivered a litter on d19.5, was 44.4% (Nodal^loxP/loxP^ n=7, Nodal^Δ/+^ n=5, Nodal^Δ/Δ^ n=9). **(B)** On average, Nodal^Δ/+^ and Nodal^Δ/Δ^ females gave birth to less pups in the first litter (Nodal^loxP/loxP^ n=6, Nodal^Δ/+^ n=3, Nodal^Δ/Δ^ n=4). **(C)** Of the five remaining plugged Nodal^Δ/Δ^ mice which did not deliver a litter 19 days after the first observed plug, there was a delay of approximately 8 days before the first parity. **(D)** The average number of litters delivered by each female during the six-month trial was reduced in both Nodal^Δ/+^ and Nodal^Δ/Δ^ mice (Nodal^loxP/loxP^ n=5, Nodal^Δ/+^ n=5, Nodal^Δ/Δ^ n=9). **(E, F)** The average number of pups delivered per litter (Nodal^loxP/loxP^ n=5, Nodal^Δ/+^ n=5, Nodal^Δ/Δ^ n=9), and total number of pups born across all litters (Nodal^loxP/loxP^ n=5, Nodal^Δ/+^ n=5, Nodal^Δ/Δ^ n=8) was significantly less in Nodal^Δ/+^ and Nodal^Δ/Δ^ females. **(G)** As maternal age increased, the number of pups per litter in Nodal^Δ/+^ and Nodal^Δ/Δ^ mice was significantly less than Nodal^loxP/loxP^ mice. **(H)** As the Nodal^Δ/+^ and Nodal^Δ/Δ^ mothers reached a later parity, less percentage of females were able to deliver a litter. By parity seven, 20% of Nodal^Δ/+^ and Nodal^Δ/Δ^ mice delivered a litter. Data shows mean ± SEM. *P<0.05, **P<0.01, ****P<0.0001.

A six-month fertility trial showed consistencies across all Nodal^Δ/Δ^ pregnancies. Females in each group were paired with a wild-type CD1 male for the duration of the breeding trial, and after confirmation of the first plug the pregnancy rate at term was found to be 44% in Nodal^Δ/Δ^ females (**Figure 2A**). The average number of pups in the first litter was significantly less in both Nodal^Δ/+^ and Nodal^Δ/Δ^ mice compared to Nodal^loxP/loxP^ controls, as previously reported for this strain (23, 24) (Nodal^loxP/loxP^ 9.33 ± 0.2, Nodal^Δ/+^ 5.67 ± 1.5, Nodal^Δ/Δ^ 5.50 ± 1.3 pups/litter) (**Figure 2B**). The 56% of plugged Nodal^Δ/Δ^ mice that did not deliver as expected on d19.5 instead delivered a litter 26-30 days from the first observed plug (**Figure 2C**). Overall, there was a reduction in the total number of litters delivered by Nodal-deficient mice across six months (Nodal^loxP/loxP^ 7.0 ± 0.3, Nodal^Δ/+^ 5.40 ± 0.5, Nodal^Δ/Δ^ 5.56 ± 0.5 litters/female) (**Figure 2D**). While the pregnancy rate of Nodal^Δ/+^ females was normal, there was still post-implantation fetal loss similar to Nodal^Δ/Δ^ pregnancies as reflected by both a significantly reduced average number of pups per litter and total number of pups delivered during the six-month trial (**Figures 2E, F**). Although Nodal^loxP/loxP^ females continued to deliver a normal-sized litter at an advanced age, the Nodal^Δ/+^ and Nodal^Δ/Δ^ mice showed an earlier decline in fertility (**Figure 2G**). By the fifth parity, the percentage of Nodal^Δ/Δ^ females that delivered a litter decreased to 67% and was further reduced to 22% by the seventh parity (**Figure 2H**). Although beyond the scope of this study, it is suggested that advanced maternal age heightens the decline in fertility due to the deletion of Nodal in the reproductive tract. In conclusion, due to the inability of most young Nodal^Δ/Δ^ mice to show signs of pregnancy after the time of implantation, it is likely that an initial subfertility can be attributed to implantation failure.

### Estrous cycling and reproductive tract morphology are unaffected in Nodal^Δ/Δ^ mice

The cyclic variation of estrogen and progesterone in the murine reproductive tract occurs during the estrous cycle and is divided into four stages: proestrus, estrus, metestrus and diestrus. Specifically, receptivity to mating and ovulation coincide during estrus and therefore is the only stage when pregnancy can occur (25, 26). To monitor progression of the estrous cycle, Nodal^Δ/Δ^ females were vaginally smeared daily for eighteen days. Each individual stage of the estrous cycle was morphologically distinct, exhibiting the characteristic ratios of nucleated epithelial cells (proestrus), cornified epithelial cells (estrus/metestrus) or leukocytes (diestrus) (**Supplementary Figure 1A**). The average cycle length was similar to Nodal^loxP/loxP^ controls (4.8 ± 0.8, Nodal^Δ/Δ^ 4.0 ± 0.2 days) (**Supplementary Figure 1B**) and the reported average of four to five days in wildtype mice (27). As a standard indicator of the regular hormonal control of estrous cycling, Nodal^Δ/Δ^ females showed a comparable plugging efficiency when mated overnight with wild-type CD1 males (Nodal^loxP/loxP^ 52%, Nodal^Δ/Δ^ 45%) (**Supplementary Figure 1C**). Since many knockout strains with uterine gland deletions or reduced morphogenesis are infertile (28–31), uteri and ovaries from d3.5 subfertile Nodal^Δ/Δ^ females were examined. Histologically, Nodal^Δ/Δ^ uteri appeared normal with abundant glands and luminal epithelial cells lining the uterine cavity (**Supplementary Figure 1D**). Furthermore, the number of corpora lutea within each ovary was counted to show successful ovulation and luteogenesis (Nodal^loxP/loxP^ 13.67 ± 2.2, Nodal^Δ/Δ^ 14.33 ± 2.2 C.L./female) (**Supplementary Figure 1E**). To summarize, reproductive tract histology, estrous cycling and corpora lutea formation were normal in females deficient in uterine Nodal expression.

### Embryos derived from Nodal^Δ/Δ^ females are abundant and viable

Implantation success is dependent on the precise synchronization between the competent blastocyst and receptive endometrium (8, 10). Embryonic abnormalities such as aneuploidy or impaired hatching from the zona pellucida are a major factor in cases of recurrent implantation failure in humans (32). Therefore, to assess oocyte quality and fertilization efficiency prior to implantation in the Nodal-deficient uterus, oocytes were isolated on d0.5. Following fertilization there was a similar number of oocytes present in the oviduct (Nodal^loxP/loxP^ 6.6 ± 1.8, Nodal^Δ/Δ^ 5.3 ± 0.8 ova/female) (**Supplementary Figure 1F**). Additionally, there was a high rate of fertilization indicated by the presence of two pronuclei or a second polar body (Nodal^loxP/loxP^ 96.9%, Nodal^Δ/Δ^ 92.5%) (**Supplementary Figure 1G**). When the oviduct and uterus were flushed on d3.5 there was no difference in the number of embryos isolated between groups (Nodal^loxP/loxP^ 5.5 ± 0.7, Nodal^Δ/Δ^ 4.8 ± 0.9 embryos/female) (**Supplementary Figure 1H**).

Embryo viability independent of the Nodal-deficient uterine environment was evaluated by transferring Nodal^Δ/Δ^ derived zygotes into the oviducts of d0.5 pseudopregnant CD1 wild-type recipients. Of 39 zygotes introduced, 24 live and healthy term pups were born (data not shown), correlating to the expected efficiency of transfer experiments (33). Conversely, the pregnancy rate was assessed following the transfer of seven wild-type blastocysts into one uterine horn of d2.5 pseudopregnant Nodal^Δ/Δ^ females (**Supplementary Figure 1I**). It was found that uteri deficient in Nodal signaling had a reduced pregnancy rate on d7.5 or complete implantation failure. The pregnancy rate of Nodal^Δ/Δ^ females after transfer with wild-type embryos was 33% in comparison to 66% of Nodal^loxP/loxP^ controls (**Supplementary Figure 1J**). Therefore, since Nodal^Δ/Δ^ derived zygotes were inherently viable and developed normally in a wild-type uterine environment, but wild-type embryos failed to implant into the Nodal^Δ/Δ^ uterus, it was conclusive that the Nodal-deficient uterus bore responsibility for implantation failure.

### Differential expression of cytokines and receptivity factors in Nodal^Δ/Δ^ uteri

**Figure 3:**
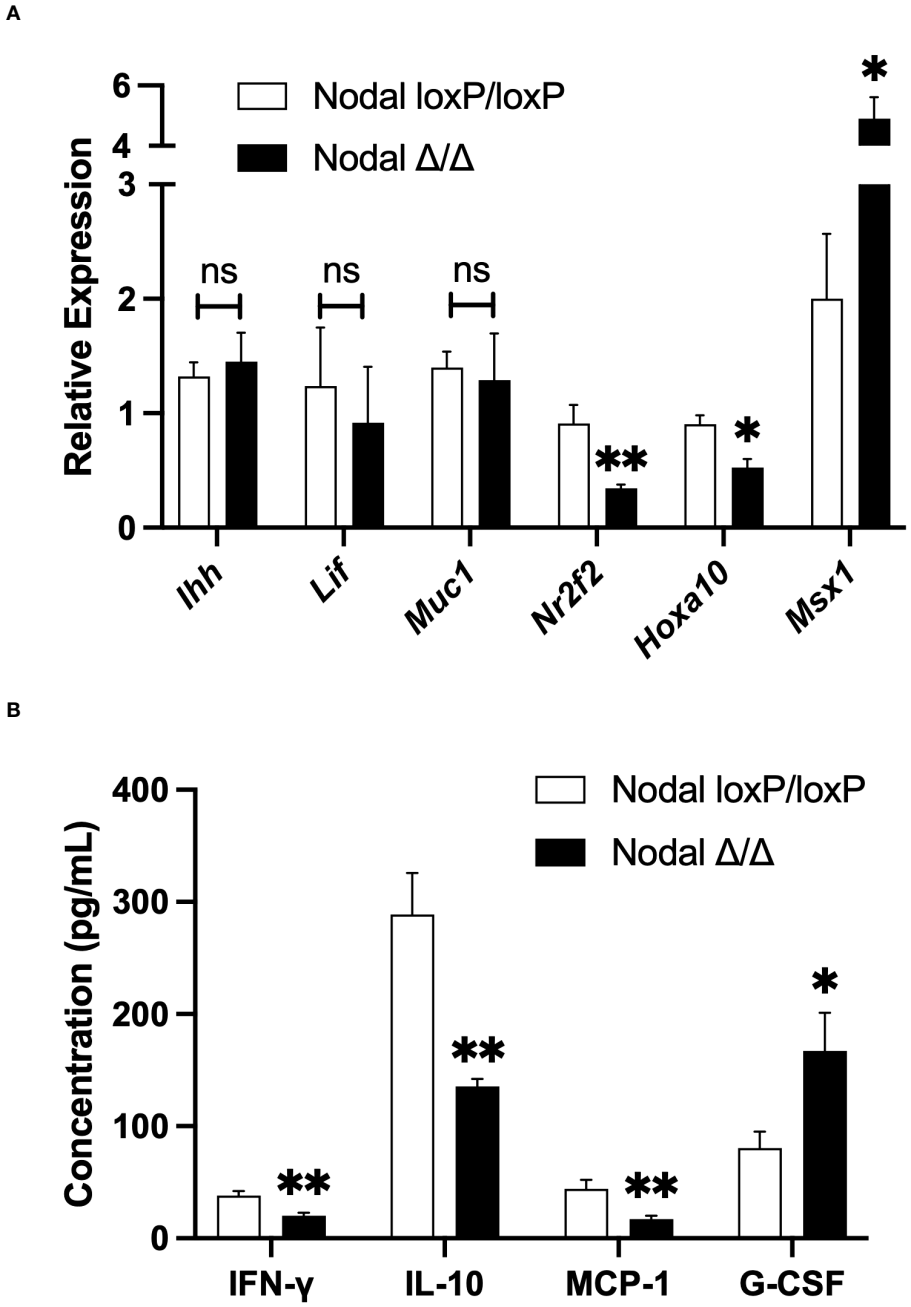
Differential expression of cytokines and receptivity factors in Nodal^Δ/Δ^ uteri. **(A)** Relative expression of genes in the d3.5 uterus by quantitative-PCR demonstrated no difference in *Ihh, Lif* or *Muc1* expression in Nodal^Δ/Δ^ uteri (n=7) compared to Nodal^loxP/loxP^ controls (n=5). However, the expression of *Nr2f2* and *Hoxa10* was significantly reduced, and *Msx1* increased in Nodal^Δ/Δ^ females. **(B)** Due to low mRNA expression, the protein abundance of inflammatory cytokines in the d3.5 uterus was determined by multiplex ELISA. IFN-γ, IL-10 and MCP-1 were significantly decreased in Nodal^Δ/Δ^ mice while G-CSF was increased (Nodal^loxP/loxP^ n=16, Nodal^Δ/Δ^ n=14). Data shows mean ± SEM. *P<0.05, **P<0.01.

The window of implantation in mice occurs between d3.5 and d4.5 as it coincides with strictly regulated changes in uterine signaling. A state of maternal endometrial receptivity is initially achieved by the transition of an estrogen-dominant proliferative state into a progesterone-responsive state. Subsequent expression of cytokines, growth factors and other signaling molecules is critical for the success of implantation (11, 12, 31). Here, expression of receptivity factors in the uterus was assessed on d3.5 by qPCR. Genes expressed downstream of ovarian hormones including *Ihh*, *Lif* and *Muc1* were similar between Nodal^Δ/Δ^ and Nodal^loxP/loxP^ females. *Nr2f2* (COUP-TFII) and *Hoxa10* were significantly reduced and *Msx1* was elevated in Nodal^Δ/Δ^ uteri (**Figure 3A**). Conditional ablation of these genes in the uterus has been previously shown to cause implantation failure or infertility in mice (28, 34–38), which confirmed the necessity for synchronous, timed gene expression during the window of implantation.

Notably, in addition to regulating the changes in gene expression preceding implantation ovarian hormones regulate the infiltration and activation of leukocytes in the uterus, which contributes substantially to the state of receptivity. In turn, the expression of many pro-inflammatory cytokines from both endometrial stromal cells and immune cells are increased prior to implantation (39, 40). On d3.5 the expression of *Il-1β*, *Il-6* and *Tnf-α* in the mouse uterus was too low to be detected by qPCR, however multiplex ELISA demonstrated no difference in protein abundance between controls and conditional knockouts (data not shown). The level of other inflammatory cytokines IFN-γ, IL-10 and MCP-1 (CCL2) were significantly decreased in Nodal^Δ/Δ^ uteri. Interestingly, G-CSF was increased in Nodal^Δ/Δ^ mice despite it being considered a pro-implantation factor (**Figure 3B**) (41). Nodal signaling appeared to have dual roles influencing both uterine gene expression and leukocyte-derived factors during the preimplantation period, the latter was of novel interest and encouraged further investigation.

### Localization and abundance of infiltrating leukocytes in the Nodal-deficient preimplantation uterus is similar to Nodal^loxP/loxP^ controls

The leukocyte population within the uterus is highly dynamic throughout pregnancy as it facilitates close interactions between the maternal endometrial and semi-allogeneic fetal cells. Precise balance of this immunological landscape (cell type, abundance and the magnitude of factors produced) is a critical determinant for initiating a healthy pregnancy. Specifically, during the preimplantation period the female reproductive tract is exposed to factors within the seminal fluid which trigger the uterine immune response to prepare for implantation (15, 42, 43). As an immunomodulatory role for maternal Nodal during late pregnancy was previously proposed (24), it was hypothesized that Nodal is also involved in the establishment of the uterine immune landscape during the preimplantation and implantation period.

**Figure 4:**
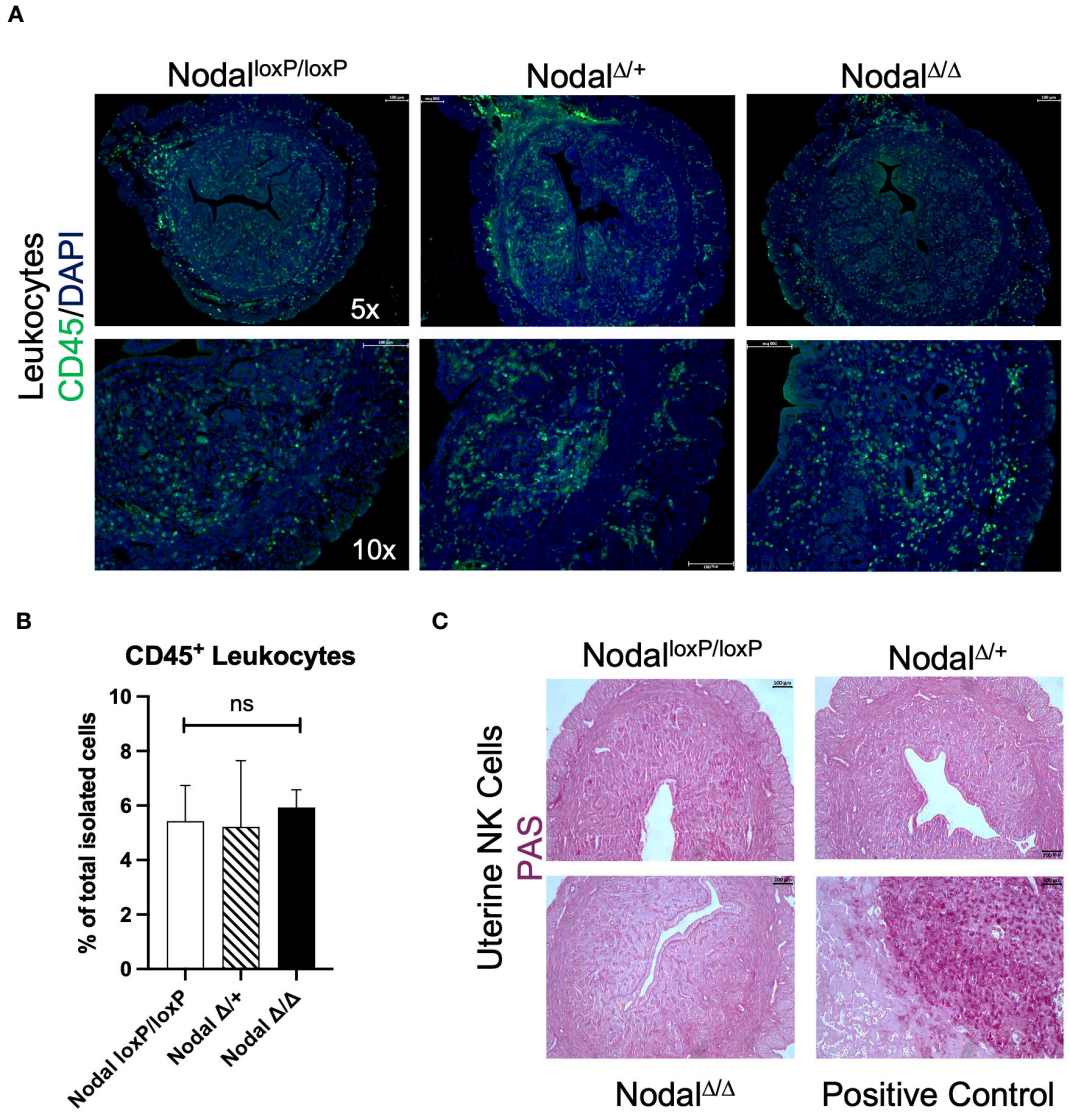
Distribution and quantity of leukocytes within the preimplantation uterus is consistent across groups. **(A)** Immunofluorescence staining revealed equal distribution of CD45^+^ leukocytes (green) within the d3.5 uterus. **(B)** Quantification of CD45^+^ cells by flow cytometry showed no difference in the percentage of leukocytes isolated (Nodal^loxP/loxP^ n=7, Nodal^Δ/+^ n=4, Nodal^Δ/Δ^ n=10). **(C)** The presence of PAS^+^ uNKs in the uterus is not detected prior to implantation (d3.5), as the expansion of this population begins during decidualization. Positive control shows the maternal decidua on d10.5 where uNKs and glycogen trophoblast cells are PAS^+^. Data shows mean ± SEM, scale bars indicate 100 µm.

Visualization of immune cells within the d3.5 uterus by immunofluorescence staining revealed CD45^+^ leukocyte populations within the endometrium, both layers of myometrium and with more visual frequency at the mesometrial pole and endometrial-myometrial junction in all groups (Nodal^loxP/loxP^, Nodal^Δ/+^ and Nodal^Δ/Δ^) (**Figure 4A**). Utilizing flow cytometry as a precise quantification method and gating strategies previously described for similar tissue types (44), it was confirmed there was no difference in the number (data not shown) or overall percentage of live, single CD45^+^ cells isolated from d3.5 uteri (Nodal^loxP/loxP^ 5.4%, Nodal^Δ/+^ 5.2%, Nodal^Δ/Δ^ 5.9%) (**Figure 4B**).

Uterine natural killer (uNK) cells are a prominent immune population during spiral artery remodeling and placentation but are uncommon in the preimplantation mouse uterus, beginning to accumulate during decidualization and peaking on d10.5 at the maternal-fetal interface (45). To definitively exclude this population in the context of murine implantation failure Periodic-acid Schiff (PAS) staining was performed. Glycoprotein-rich PAS^+^ uNKs were absent from all d3.5 uterine sections as expected (**Figure 4C**) but abundant within the d10.5 implantation site in addition to glycogen-containing PAS^+^ trophoblast cells (46). Since the total number of CD45^+^ cells within the preimplantation uterus was unaffected in Nodal^Δ/Δ^ mice, a more meaningful determinant of maternal immune activation prior to implantation would be to consider the composition of the myeloid and lymphocyte subpopulations.

### Significant increase in the proportion of neutrophils and macrophages in d3.5 Nodal^Δ/Δ^ uteri

In response to seminal TGFβ, neutrophils infiltrate the uterus to clear excess sperm, fluid and restore microbial balance (47). Macrophages and dendritic cells are also recruited and present antigens to naïve T cells in the draining lymph node, eventually establishing a residential uterine regulatory T cell (Treg) population and maternal tolerance (43, 48, 49). Alternatively, pro-inflammatory macrophages mediate the controlled breakdown of the uterine epithelium and tissue remodeling during blastocyst attachment (50). Depletion of CD11b^+^ macrophages during early pregnancy was reported to cause complete implantation failure in mice (51), while an excess of inflammatory macrophages has been implicated in cases of recurrent implantation failure and spontaneous abortion in humans (52–55). Therefore, a balanced macrophage reaction is essential for initiating the functional immune response at implantation.

**Figure 5:**
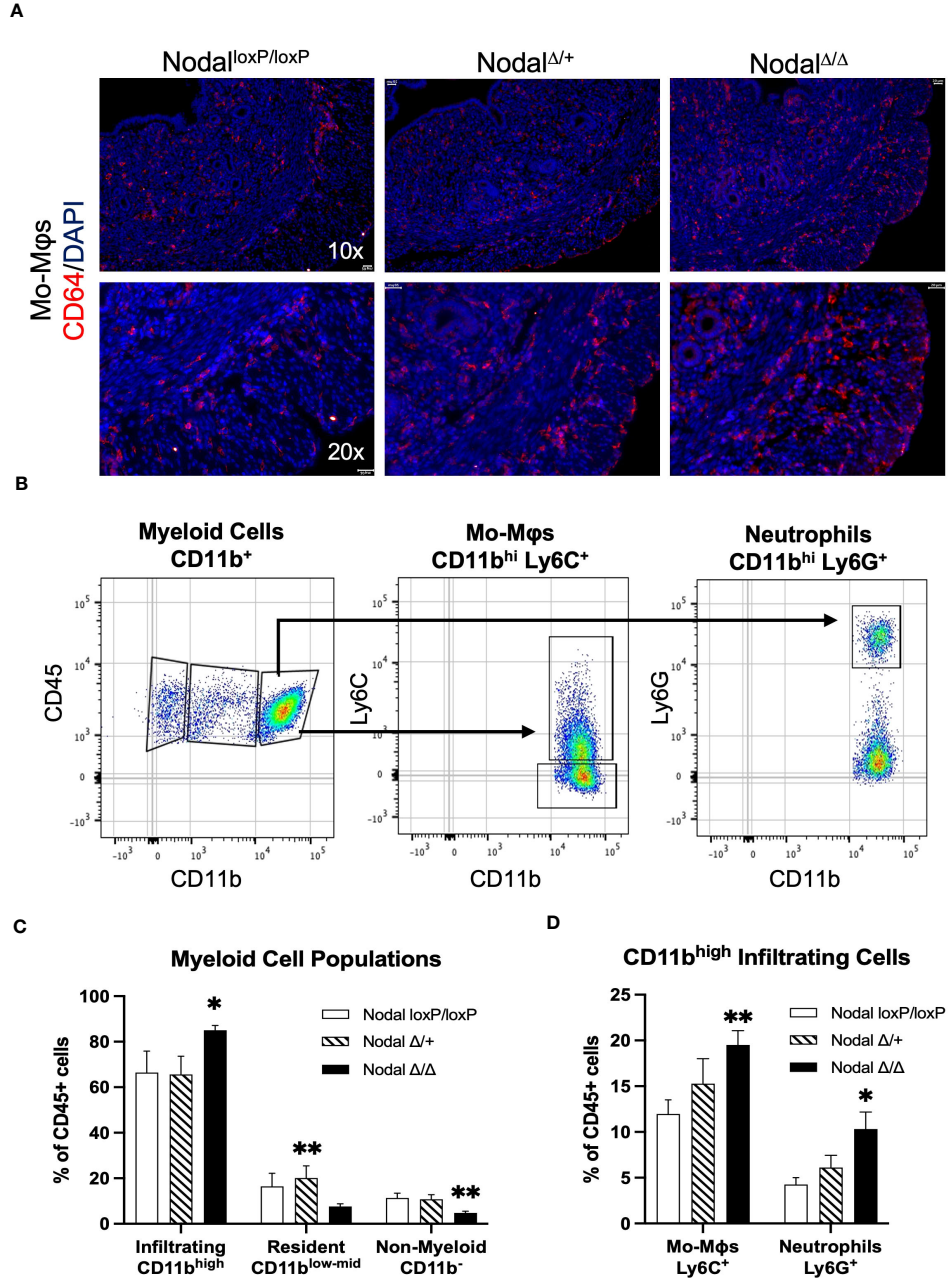
Increased abundance of CD11b^high^ myeloid cells in the Nodal^Δ/Δ^ preimplantation uterus. **(A)** Immunofluorescence staining shows similar localization but higher visual frequency of CD64^+^ immune cells (monocytes and macrophages) within the d3.5 uteri of Nodal^Δ/Δ^ females compared to Nodal^loxP/loxP^ and Nodal^Δ/+^ females. Scale bars indicate 100 µm. **(B)** Myeloid-derived cells in the d3.5 uterus were gated from the CD45^+^ live cell population based on expression of CD11b, and then with further lineage markers of Ly6C (monocyte-derived macrophages, Mo-Mϕ) and Ly6G (neutrophils). **(C)** Quantification of CD11b^+^ populations in the d3.5 preimplantation uterus (Nodal^loxP/loxP^ n=7, Nodal^Δ/+^ n=4, Nodal^Δ/Δ^ n=9) showed a significant increase in the number of infiltrating myeloid cells in Nodal^Δ/Δ^ mice, and a decrease in the number of non-myeloid CD11b^-^ leukocytes. **(D)** Both Ly6C^+^ macrophages and Ly6G^+^ neutrophils are increased in Nodal^Δ/Δ^ uteri. Data shows mean ± SEM. *P<0.05, **P<0.01.

To determine the composition of the myeloid population residing in the preimplantation uterus, immunofluorescence staining was performed using CD64 to identify monocytes and macrophages (**Figure 5A**). CD64^+^ cells were observed in all layers of the d3.5 uterus (endometrium, myometrium and perimetrium) in all groups. Although the localization of the CD64^+^ cells within the uterus was similar between groups, the frequency of these cells seemed much higher in the d3.5 Nodal^Δ/Δ^ uteri specifically towards the uterine periphery in comparison to Nodal^loxP/loxP^ and Nodal^Δ/+^ mice.

Isolated cell suspensions from d3.5 flushed Nodal uteri were stained using a panel of antibodies designed for classifying general leukocyte populations. Using CD11b as a marker for myeloid cell migration and adhesion (44, 56), three populations were gated as CD11b^-^ (non-myeloid), CD11b^low-mid^ (resident) or CD11b^high^ (infiltrating) (**Figure 5B**) using flow cytometry. The non-myeloid CD11b^-^ population (which includes the CD3^+^ and CD19^+^ lymphocytes) was significantly less in Nodal^Δ/Δ^ preimplantation uteri (4.8%, Nodal^loxP/loxP^ 11.4%, Nodal^Δ/+^ 10.8% of leukocytes) (**Figure 5C**). Intermediary CD11b^low-mid^ residential myeloid cells were found to be more numerous only in Nodal^Δ/+^ females when compared to knockout mice (20.1%, Nodal^loxP/loxP^ 16.4%, Nodal^Δ/Δ^ 7.6% of leukocytes) (**Figure 5C**). The CD11b^high^ infiltrating cells were substantially increased from 66.4% in Nodal^loxP/loxP^ mice to 85.0% in Nodal^Δ/Δ^ mice and was determined to be the major leukocyte population present prior to implantation (**Figure 5C**). Further analysis of the infiltrating CD11b^high^ leukocytes using Ly6C, a marker for monocyte-derived pro-inflammatory macrophages (Mo-Mϕ) (57), revealed a considerable increase in macrophage abundance with almost double the proportion observed in Nodal^Δ/Δ^ uteri (Nodal^loxP/loxP^ 12.0%, Nodal^Δ/+^ 15.3%, Nodal^Δ/Δ^ 19.5% of leukocytes). Similarly, the amount of Ly6G^+^ neutrophils was doubled in Nodal^Δ/Δ^ mice compared to Nodal^loxP/loxP^ controls (Nodal^loxP/loxP^ 4.3%, Nodal^Δ/+^ 6.1%, Nodal^Δ/Δ^ 10.3% of leukocytes) (**Figure 5D**). Evidentially, the myeloid response in Nodal^Δ/Δ^ uteri was overwhelmingly increased in magnitude compared to Nodal^loxP/loxP^ mice.

### Nodal^Δ/Δ^ females lack FOXP3^+^ regulatory T cells during the preimplantation period

Counteractive to the macrophage response is the activity of Tregs which function by promoting the immunosuppressive maternal uterine environment required for implantation of the semi-allogeneic embryo (43, 49). This is mediated through the production of cytokines that polarize anti-inflammatory “M2” macrophages, the regulation of T effector cell types and the support of maternal vascularization (19). Current research has emphasized the role of Tregs during implantation as insufficiencies in both overall number and function, with a subsequent increase in T effector types, has been observed in various reproductive pathologies (18, 19, 58, 59).

**Figure 6:**
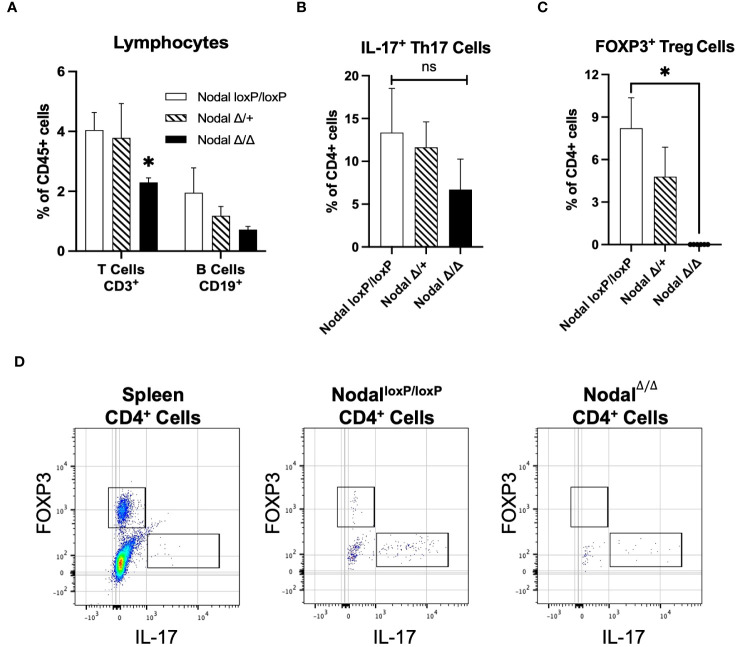
Significant reduction in the frequency of T cells accompanied by a lack of Treg cells in the preimplantation uterus of Nodal^Δ/Δ^ females. **(A)** Quantification of CD3^+^ T cell populations in the preimplantation uterus (Nodal^loxP/loxP^ n=7, Nodal^Δ/+^ n=4, Nodal^Δ/Δ^ n=10) showed a significant decrease in the number of T cells in Nodal^Δ/Δ^ mice, and a trending decrease in the number of CD19^+^ B cells. **(B)** Further characterization of the CD3^+^ CD4^+^ population showed a non-significant but trending reduction in the percentage of IL-17^+^ Th17 cells in Nodal^Δ/Δ^ uteri (Nodal^loxP/loxP^ n=10, Nodal^Δ/+^ n=8, Nodal^Δ/Δ^ n=8). **(C)** Analysis of the FOXP3^+^ Treg population showed that although rare in Nodal^loxP/loxP^ and Nodal^Δ/+^ females, a clear population could be defined. However, there was a complete absence of FOXP3^+^ Treg cells in the Nodal^Δ/Δ^ d3.5 uterus. **(D)** Gating strategy to determine FOXP3^+^ and IL-17^+^ CD4^+^ populations in the preimplantation uterus (concatenated) based on marker expression in the spleen. Data shows mean ± SEM. *P<0.05.

It was indicated that the CD11b^-^ population was affected in Nodal^Δ/Δ^ females (**Figure 5C**), so CD3 and CD19 were first used to characterize the uterine lymphocyte populations. The CD3^+^ T cell population was significantly reduced in the d3.5 Nodal^Δ/Δ^ uteri, amounting to 2.3% of total leukocytes and about half the number present in controls (Nodal^loxP/loxP^ 4.0%, Nodal^Δ/+^ 3.8% of leukocytes). The portion of CD19^+^ B cells was not significantly different across groups but trended towards a decrease in the Nodal^Δ/Δ^ females (**Figure 6A**). CD3^+^ T cells could be further classified into subpopulations of activated T effectors based on the expression of CD8 (cytotoxic T) or CD4 (T helper, Th). There was no difference in the total percentage of CD8^+^, CD4^+^ or the subset of CD4^+^ IFN-*γ* ^+^ (Th1) cells across groups (data not shown). CD4^+^ IL-17^+^ (Th17) cells had no statistical difference but showed a strong trending decrease in Nodal^Δ/Δ^ uteri (Nodal^loxP/loxP^ 13.4%, Nodal^Δ/+^ 11.6%, Nodal^Δ/Δ^ 6.7% of CD4^+^ leukocytes) (**Figure 6B**). Strikingly, the CD4^+^ FOXP3^+^ Treg population, which is necessary for maternal tolerance during implantation, was completely non-existent in the d3.5 uterus of Nodal^Δ/Δ^ females (**Figures 6C, D**). Although rare and in low abundance, Nodal^loxP/loxP^ and Nodal^Δ/+^ mice had obvious CD4^+^ FOXP3^+^ populations (Nodal^loxP/loxP^ 8.2%, Nodal^Δ/+^ 4.8% of CD4^+^ leukocytes) (**Figure 6D**). The absence of FOXP3^+^ Tregs in uteri of Nodal^Δ/Δ^ females during the preimplantation period provided justification for the observed implantation failure. Therefore, it is proposed that uterine Nodal signaling during the preimplantation period is important for the development of the FOXP3^+^ Treg population and the establishment of maternal immunotolerance to pregnancy.

## Discussion

Within the last decade very few studies have investigated the function of Nodal during reproduction beyond its usual role in embryonic development. In comparison to other TGFβ superfamily members uterine Nodal expression is not well characterized, however the initiation of Nodal expression shortly after mating in the adult mouse uterus provided a strong indication for its role during pregnancy (20). The generation of the conditional knockout Nodal^Δ/Δ^ strain proved that Nodal was necessary for successful reproduction since all Nodal-deficient females showed severe subfertility, seen as a reduced pregnancy rate at term and smaller litter size (23). Although only later stages of gestation were considered, it was revealed that uterine Nodal expression promoted an anti-inflammatory state before parturition (24). Acknowledging the dynamics of the maternal immune response to pregnancy and how dysregulation at the earliest stages could result in later pathological pregnancy complications, it was hypothesized that uterine Nodal contributed to the immunotolerant environment established during the preimplantation period.

Despite normal preimplantation processes and viable embryos (**Supplementary Figure 1**), Nodal^Δ/Δ^ females were shown to have a 50% implantation failure rate (**Figures 1A, B**). Similarly, previous *in vivo* gene transfer experiments of exogenous Lefty (the inhibitor of Nodal signaling) into the preimplantation uterus of wild-type CD1 females significantly decreased implantation efficiency. Both the use of a Lefty retroviral expression vector system or liposome-mediated introduction of a Lefty expression vector showed either a reduced number of embryos on d9.5 or complete implantation failure (60). Nodal^Δ/Δ^ females demonstrated a very similar phenotype to both Lefty overexpression experiments, therefore either directly knocking out Nodal from the uterus or overexpressing the inhibitor Lefty throughout the preimplantation period can lead to implantation failure. These independently generated results provided an intriguing correlation given the intricacies of the Nodal signaling pathway and crosstalk with other TGFβ superfamily members. Many mouse models with conditional genetic deletions of TGFβ family ligands, receptors or signaling components share reproductive phenotypes throughout gestation, but the precise signaling pathways of each factor remains difficult to interpret due to redundancies and potential compensatory functions within the superfamily (21, 61, 62). This could explain implantation success for a portion of the Nodal^Δ/Δ^ females. Outside of the TGFβ superfamily, TGFβ signaling has been shown to regulate the Wnt and hedgehog pathways which are fundamental in preparing the uterus for embryo implantation (8, 62). Wnt and hedgehog pathways are upstream of the endometrial receptivity factors *Nr2f2* (COUP-TFII), *Hoxa10* and *Msx1* that were shown to be dysregulated during the window of implantation in Nodal^Δ/Δ^ mice (**Figure 3A**). Although beyond the scope of this study, it is not inconceivable that Nodal too is involved in the complexities of uterine receptivity through perhaps an unknown interaction with TGFβ signaling or Wnt/hedgehog pathways. In either case, uninterrupted uterine Nodal signaling is imperative for efficient embryo implantation.

The continued housing of breeding pairs allowed for the observation of Nodal^Δ/Δ^ females natural mating behaviors. Interestingly, the plugged Nodal^Δ/Δ^ females that experienced complete implantation failure after the first mating were eventually successful in delivering a litter, however delayed 7-11 days when compared to those that delivered on time after the first mating (**Figure 2C**). Nodal^Δ/Δ^ mice were classified as having delayed pregnancy as opposed to delayed implantation as the pregnancy rate did not improve at later timepoints of dissection (**Figure 1A**). Essentially, if mating did not result in fertilization or implantation, the corpora lutea regressed and the next estrus cycle followed after 10-12 days (63, 64). Sustained pairing of mice permitted a second mating within this period and ultimate success of the second Nodal^Δ/Δ^ pregnancy. Repeated exposure to seminal fluid over the course of multiple mating cycles with the same partner in both mouse and human studies was shown to increase the capacity of the maternal immune response to tolerate future pregnancies, in addition to reducing the risk of developing preeclampsia (43, 65, 66). Furthermore, *in vitro* fertilization treatments co-treated with seminal fluid during the time of embryo transfer significantly increased the rate of clinical pregnancy (67), confirming the importance of paternal (fetal) antigen conditioning within the uterus for maternal tolerance. Together, the occurrence of implantation failure, delayed pregnancy or further reproductive challenges during mid-gestation for those Nodal^Δ/Δ^ females with implantation success overwhelmingly supported the argument for a dysregulated maternal immune response in the Nodal-deficient preimplantation uterus.

Inflammatory environments during pregnancy can be classified based on the dominance of either pro-inflammatory, classical “M1” or anti-inflammatory, alternatively activated “M2” macrophages. Though this is an oversimplification of concepts and perhaps more representative of *in vitro* conditions (57), the dynamic polarization of macrophages in response to the *in vivo* uterine microenvironment at different stages of gestation is imperative for a healthy pregnancy (68, 69). MCP-1 (CCL2) is a driver of myeloid cell recruitment into the uterus, and in response to seminal factors during mating MCP-1 expression increases during the preimplantation period and window of implantation. This is coupled with an increase in the number of M1-polarized infiltrating macrophages (70). Although MCP-1 was decreased in the d3.5 Nodal^Δ/Δ^ uteri (**Figure 3B**), the proportion of CD11b^high^ myeloid cells (**Figure 5C**) and CD11b^high^ Ly6C^+^ infiltrating pro-inflammatory macrophages (**Figures 5A, D**) was almost doubled. While the M1 state is more prevalent at implantation, the magnitude of the inflammatory response still needs to be appropriate since a maternal environment that is excessively pro-inflammatory and hostile would not be favorable for implantation. Therefore, perhaps reduced expression of MCP-1 with an increase of G-CSF (**Figure 3B**) before implantation in Nodal^Δ/Δ^ females was a mechanism to preserve the integrity of the uterus in response to the overwhelming infiltration of pro-inflammatory macrophages. These findings during early pregnancy in Nodal^Δ/Δ^ mice mirror those from previous studies of later pregnancy, as increased infiltration of decidual macrophages and expression of pro-inflammatory cytokines were seen in the Nodal^Δ/+^ uterus before parturition and led to the increased susceptibility for LPS-induced preterm labor. Complementary *in vitro* experiments were performed using both bone marrow-derived macrophages and RAW264.7 cell lines, where upon pre-treatment with recombinant Nodal protein (rNodal) before LPS the level of pro-inflammatory cytokines IL-1β, IL-6 and TNF-α was significantly reduced (24), corroborating other reports of rNodal polarizing primary mouse macrophages into an M2 state (71). Together, there is compelling evidence that uterine Nodal is an anti-inflammatory mediator of macrophage responses throughout pregnancy. Although there was no difference in the level of pro-inflammatory cytokines IL-1β, IL-6 and TNF-α on d3.5 (data not shown), this could be due to the inclusion of both structural uterine cells and leukocytes in the samples and is a limitation of this study. More definitive relationships between specific immune populations and their secreted factors in a Nodal-deficient environment could be proven by cell sorting prior to analysis, single-cell sequencing or flow cytometry panels with additional M1/M2 intracellular cytokine markers. The dendritic cell population which shares similar but distinct functions to macrophages during early pregnancy should also be addressed in the Nodal^Δ/Δ^ model. Unfortunately, few studies consider the mouse preimplantation innate myeloid response, as more emphasis has been placed on understanding post-implantation decidualization, placentation and parturition processes. Current research in reproductive immunology concerning implantation and infertility has instead focused on the adaptive T cell responses. As the CD11b^-^ (**Figure 5C**) and CD3^+^ (**Figure 6A**) was significantly less in d3.5 Nodal^Δ/Δ^ uteri, there was indication that the responding lymphocyte population was also impacted by the deletion of uterine Nodal.

Mild inflammation generated by stromal and myeloid cells within the uterus recruits and induces CD4^+^ T cells into the proper effector phenotype required for implantation, including Th1, Th17 and Treg responses (72). IFN-γ is the major cytokine produced by activated Th1 cells which contributes to the dominant pro-inflammatory state at implantation. The protein level of uterine IFN-γ was significantly decreased in Nodal^Δ/Δ^ females on d3.5 (**Figure 3B**) however no difference was observed in the number of CD4^+^ IFN-γ^+^ Th1 cells (data not shown). Excessive Th1 immunity is correlated with recurrent implantation failure, recurrent pregnancy loss and miscarriage (73, 74) and was expected to be prevalent in the Nodal^Δ/Δ^ implantation failure model. Alternatively, while the myeloid source of IFN-γ during implantation has been debated (75) some studies have shown that it can be produced by macrophages and assist in Th1 polarization (76), so maybe differential levels of IFN-γ in the preimplantation Nodal^Δ/Δ^ uterus implicates the immunoreactivity of M1 macrophages. IL-17 producing Th17 cells have been shown to be elevated in the peripheral blood of women with recurrent implantation failure and pregnancy loss (58, 77). Similar to the Th1 response, this was expected to be elevated in Nodal^Δ/Δ^ females but was instead almost significantly decreased (**Figure 6B**). Conversely, Tregs function by limiting excessive inflammation while suppressing these effector T cell responses to fetal antigens and sustaining maternal tolerance (19). Reduced levels of uterine IL-10 (**Figure 3B**) could be due to the absence of a FOXP3^+^ Treg population in d3.5 Nodal^Δ/Δ^ uteri (**Figures 6C, D**). Overall, Treg deficiencies have been causal in numerous mouse and human studies of infertility (19, 78). The interplay and plasticity of Th1, Th17 and Treg lineages from the naïve CD4^+^ state in the presence or absence of Nodal signaling encourages further investigation.

It is unclear if the lack of uterine FOXP3^+^ Tregs on d3.5 in Nodal^Δ/Δ^ females is the direct result of failed induction of naïve CD4^+^ T cells at any point during the preimplantation period, or possibly just the loss of proliferation and maintenance of these Tregs after an initial wave of recruitment into the uterus. It should be noted that the lack of uterine Nodal was shown to effect only the FOXP3^+^ Tregs, and any impact on the FOXP3^-^ population is still undetermined. Since the number of Tregs in the preimplantation mouse uterus is very limited, complementary *in vitro* experiments would also be beneficial. It has been well established that TGFβ is an inducer of Tregs during normal immune functions and pathological conditions (79). Activin A (a TGFβ superfamily member) was previously shown to promote the conversion of CD4^+^ CD25^-^ T cells into induced FOXP3^+^ Tregs in a dose-dependent manner with TGFβ *in vitro.* Although Activin A alone was able to induce a moderate level of conversion, the overall effect was additive when using Activin A and low concentrations of TGFβ1 together (80). Interestingly, Activin A and Nodal share the same membrane receptor (ALK4) and activate intracellular SMAD2/3 signaling pathways. Since redundancy and interactions between TGFβ superfamily members was previously highlighted (21), it is now hypothesized that Nodal acts similarly to Activin A by directly promoting the induction of Tregs *in vitro*. Alternatively, indirect mechanisms of Nodal signaling supporting maternal tolerance during pregnancy could be through the polarization of macrophages as previously reported *in vitro* (24, 71). Current work is therefore focused on *in vitro* and *ex vivo* assays to determine the precise contribution of Nodal signaling to the regulation and interaction of decidual macrophage and T cell populations. Results from these ongoing assays would elevate *in vivo* data and identify a new role for Nodal in the support of maternal tolerance during the preimplantation period, and this mechanism could be relevant to the later gestational phenotypes seen in Nodal^Δ/Δ^ mice.

Finally, from human studies involving non-pregnant women it was shown that Nodal and its inhibitor Lefty had opposing phase-dependent expression patterns throughout the menstrual cycle. Uterine Nodal expression steadily increased throughout the early- to late-proliferative phases and into the early-secretory phase but dropped during the mid-secretory phase and menses. This was particularly interesting as the shift between Nodal or Lefty dominance occurred during the mid-secretory phase when the uterus was receptive to implantation (81, 82). Indeed, Lefty misexpression has been implicated in cases of unexplained infertility (83). More recently, the association between uterine inflammatory environments and Nodal expression in later human reproductive pathologies was proposed. In a Dutch cohort of familial, intrauterine growth restriction-complicated preeclampsia the Nodal “H165R” single nucleotide polymorphism (SNP) was present in all affected women and reduced Nodal activity by 50% (84). This same SNP was also a significant risk factor for preterm labor in a separate retrospective study, but only if there was existing placental inflammation (defined as membrane inflammation, funisitis and/or umbilical cord vasculitis). Similarly, other Nodal SNPs were a risk factor for delivering preterm if the woman had bacterial vaginosis (85). Evidentially, the connection between uterine Nodal signaling and inflammatory environments during mouse pregnancy is conserved in human pregnancies. Based on the main findings from the Nodal^Δ/Δ^ mouse model in combination with evidence of disrupted Nodal-Lefty signaling in women with fertility complications, current work is concentrated on the potential association between Nodal SNPs and immune profiles of women with recurrent implantation failure. Broadly, the intriguing relevance of Nodal signaling on the modulation of inflammatory states could be applicable to other clinical cases beyond reproduction.

In conclusion, initial dysregulation of the maternal immune landscape during the preimplantation period of Nodal^Δ/Δ^ females has negative impacts on the establishment and progression of pregnancy. Here, during the preimplantation period the overabundance of CD11b^high^ Ly6C^+^ pro-inflammatory macrophages combined with the absence of CD4^+^ FOXP3^+^ Tregs in Nodal-deficient mice was detrimental to embryo implantation in 50% of cases. This suggests other mechanisms are involved, and the extent of maternal Nodal signaling in the preparation for implantation remains to be defined. A poor maternal immune response during the preimplantation period is predicted to amplify over the course of gestation and complicate later processes like placentation and sustained tolerance that are extremely dependent and vulnerable to immunomodulation. Therefore, it is proposed that uterine Nodal signaling during the preimplantation period has a novel role in supporting the initiation of maternal tolerance to pregnancy, and its dysregulation should be emphasized as a potential contributor to cases of female infertility and recurrent implantation failure.

## Materials and methods

### Generation and maintenance of Nodal conditional heterozygous and knockout mice

Experimental protocols in this study were in accordance with regulations established by the Canadian Council on Animal Care and were reviewed by the Animal Care Committee of the McGill University Health Centre. Animals were housed according to the rodent husbandry standard operating procedure #508 of the Animal Resources Division at the Research Institute of the McGill University Health Centre. The generation of these mice has been previously described (23). Mice with loxP sites flanking exons 2 and 3 of the *Nodal* gene (Nodal^loxP/loxP^) on a mixed background were kindly donated by E.J. Robertson (University of Oxford) (86). Progesterone receptor (*Pgr)*-Cre females (*Pgr* ^Cre/+^) on a C57BL6/129 background were generously provided by F.J. DeMayo and J.P. Lydon (Baylor College of Medicine) (87). Both strains have been previously reported to demonstrate normal fertility, and *Pgr* ^Cre/+^ mice are a standard model to investigate uterine-specific gene functions (36, 88). Nodal^loxP/loxP^ and *Pgr* ^Cre/+^ strains were crossed, and the offspring were genotyped by PCR. In this study, 8–12-week-old *Nodal* floxed control (Nodal^loxP/loxP^/*Pgr* ^+/+^ - denoted Nodal^loxP/loxP^), *Nodal* conditional heterozygous (Nodal^loxP/+^/*Pgr* ^Cre/+^ - Nodal^Δ/+^) and *Nodal* conditional knockout (Nodal^loxP/loxP^/*Pgr* ^Cre/+^ - Nodal^Δ/Δ^) females were used as experimental mice.

### Fertility trial

To assess the pregnancy rate of Nodal^loxP/loxP^, Nodal^Δ/+^ and Nodal^Δ/Δ^ mice, eight-week-old virgin, littermate females from each group were mated and housed with a wild-type CD1 male. The presence of a copulatory plug indicated successful mating and it was considered day 0.5 of pregnancy (d0.5). Females were monitored at the expected time of delivery (d19.5) for the birth of a litter to determine initial pregnancy rate as well as the number of pups delivered. Each female was continuously housed with a paired wild-type male so normal fertility data could be recorded for exactly six months from the date of the first plug.

### Embryo flushing

Oviducts and uterine horns, separated from the ovaries and cervix of Nodal^loxP/loxP^, Nodal^Δ/+^ and Nodal^Δ/Δ^ females on d3.5 were dissected. Oviduct and uterine horns were flushed from both ends using Hanks’ Balanced Salt Solution (HBSS) into a tissue culture dish. The isolated embryos were counted and staged. Flushed uterine horns were used for DNA, RNA, and protein extraction or enzymatic digestion to generate single cell suspensions.

### Vaginal smearing and estrous staging

Vaginal smears were collected daily at 10:00 am for eighteen days from five Nodal^loxP/loxP^ and Nodal^Δ/Δ^ females. The vaginal cavity was rinsed with PBS and wet-mount slides were immediately prepared and examined under a light microscope. The estrous stage was determined by the relative ratio of cells observed: diestrus; primarily leukocytes, proestrus; only nucleated epithelial cells, estrus; predominately large cornified epithelial cells, metestrus; moderate leukocytes with remaining cornified cells evident (89). Slides were dried overnight to promote cell adherence, washed, counter-stained with Nuclear Fast Red, dehydrated with an increasing ethanol gradient and mounted for imaging.

### Embryo transfer

Nodal^loxP/loxP^ and Nodal^Δ/Δ^ females were mated with wild-type CD1 males and the oviducts from plugged females were dissected on d0.5 into M2 media. The cumulus oocyte complexes were released by gently tearing open the infundibulum. Ova were isolated by brief incubation in hyaluronidase (300 μg/mL) to digest the cumulus mass, and fertilization of individual ova was determined by the presence of two pronuclei or a second polar body. Fertilized zygotes from either Nodal^Δ/Δ^ or Nodal^loxP/loxP^ females were pooled and 19-20 zygotes were transferred into the oviducts of anesthetized, d0.5 pseudopregnant CD1 recipient females. Recipients were allowed to recover, and pregnancy was monitored until parturition when delivered pups were quantified.

The reciprocal transfer to determine implantation efficiency of Nodal^Δ/Δ^ or Nodal^loxP/loxP^ uteri was achieved by mating the respective females with vasectomized CD1 males. On day 2.5 of pseudopregnancy, blastocysts generated from naturally mated, wild-type CD1 females (d3.5) were transferred directly into one uterine horn of anesthetized Nodal^Δ/Δ^ or Nodal^loxP/loxP^ recipients. The contralateral horn was utilized as a negative control by injecting the same volume of KSOM embryo media. The experimental and control females were allowed to recover, and uteri were removed on d7.5 to determine the conceptus site number and calculate implantation efficiency.

### Tissue processing, paraffin embedding, and sectioning

Paraffin embedding and tissue histology methods were employed as previously described (90). Briefly, dissected samples were collected in PBS and fixed in 10% neutral buffered formalin for a minimum of 48 hours at 4 °C. Samples were dehydrated in increasing concentrations of ethanol and cleared in xylene before embedded into paraffin wax (Tissue Tek). Blocks were slowly solidified on a cold plate for one hour before transfer to a -20 °C freezer until sectioning. Using the Leica RM2145 microtome, 7 μm transverse sections were cut and mounted onto Fisher Superfrost plus slides and dried. Slides were either used for immunofluorescence, PAS staining or Hematoxylin and Eosin staining (90). To count absolute corpora lutea number serial sections of the complete ovary were prepared and counter-stained with Nuclear Fast Red.

### Immunofluorescence

Uteri from d3.5 Nodal^loxP/loxP^, Nodal^Δ/+^ and Nodal^Δ/Δ^ females were dissected, fixed, embedded and sectioned as described above. Immunofluorescence staining was conducted as previously described (90). Incubation with the primary antibody CD45 (BioLegend Cat. No. 103102, 1:100) or CD64 (BioLegend Cat. No. 161002, 1:100) occurred at 4 °C overnight. Following washes with 0.1% TBS-Tween 20, slides were incubated with the appropriate secondary antibody; donkey α-rat Alexa Fluor 594 (Invitrogen Cat. No. A21209, 1:300) or goat α-rat Alexa Fluor 488 (Invitrogen Cat. No. A11006, 1:300) and DAPI (1:500) for two hours at room temperature. Slides were washed and mounted.

### PAS staining

Slides from d3.5 uteri were stained using Periodic-acid Schiff (PAS) by the Histopathology Platform at the Research Institute of the McGill University Health Centre following standard protocols without counterstaining.

### Quantitative PCR

Isolated tissues were immediately frozen on dry ice and stored at -80 °C. Total RNA extraction was conducted using Trizol (Invitrogen Cat. No. 15596018) and the RNeasy Mini Kit (Qiagen Cat. No. 74104). QuantiTect Reverse Transcription Kit (Qiagen Cat. No. 205311) was used for cDNA synthesis. Quantitative PCR (qPCR) was performed using the Rotor-Gene SYBR Green PCR Kit (Qiagen Cat. No. 204074) following the manufacturer’s protocol. Each biological replicate was performed in technical triplicate. Samples were run on the Corbett Rotor-Gene 6000 thermocycler and analyzed using the Rotor-gene 6000 software. The primers used are listed in **Supplementary Table 1** and were designed using the NCBI primer-blast tool. The relative expression of the gene of interest was calculated by the ΔΔCt method, where fold changes in gene expression were normalized to an internal control (*Gapdh*) and relative to one sample (calibrator). Each qPCR run underwent melt curve analysis to confirm the presence of one peak.

### Protein extraction and multiplex ELISA

Uterine horns from d3.5 mice were flushed as described before. Samples were immediately frozen on dry ice and stored at -80 °C until protein extraction. Tissue was weighed and homogenized in 1 mL lysis buffer per 50-100 mg tissue (20 mmol/L Tris-HCl pH 7.5, 150 mmol/L NaCl, 0.05% Tween-20, 1x AEBSF protease inhibitor (Sigma Cat. No. 30827-99-7)). After centrifugation, the total protein in the supernatant was quantified using the BCA Protein Assay Kit (Thermo Scientific Cat. No. 23227) and Tecan Infinite M200 Pro plate reader. Protein concentrations were determined by comparing absorbance values to a standard BSA curve and equalized to 2 mg/mL prior to the assay. The concentrations of G-CSF, GM-CSF, IL-1β, IL-6, IL-10, IL-15, IFN-γ, LIF, M-CSF, MCP-1 (CCL2), MIP-1β (CCL4) and TNF-α were measured by multiplex ELISA using a custom Milliplex MAP Mouse Cytokine/Chemokine Magnetic Bead Panel (Millipore Cat. No. MCYTOMAG-70K) according to the manufacturer’s instructions. Each biological replicate was performed in technical duplicate. The multiplex plate was read on the Luminex 200 System (Millipore) and the data was analyzed with the Belysa (Millipore Version 1.1.0) and Microsoft Excel software, fitting absorbance values to a standard curve.

### Flow cytometry

Flushed d3.5 uterine horns from each group were weighed and digested by Liberase TM (Roche Cat. No. 5401119001) in HBSS supplemented with 2% FBS (25 μg Liberase per 0.1 g of tissue). Digestion occurred for 45 minutes at 37 °C, with agitation every 10 minutes, and products were filtered through a 70 μm cell strainer. To generate a single-cell suspension from the spleen, tissue was pushed through the cell strainer using a syringe plug and treated with 5 mL ACK buffer pH 7.2 (150 mm/L NH_4_Cl, 10 mm/L KHCO_3_, 0.1 mm/L Na_2_EDTA) for 30 seconds. 5 mL cold PBS was added to stop the lysis reaction. When necessary for intracellular cytokine staining, cells were stimulated with Cell Activation Cocktail (phorbol-12-myristate 13-acetate and ionomycin, BioLegend Cat. No. 423301) following manufacturers protocol for 4 hours in HBSS/2% FBS. Monensin (BioLegend Cat. No. 420701) transport inhibitor was added during the last hour of incubation. 1 million cells/sample were blocked using the FcγR antibody for 10 minutes (BD Biosciences Cat. No. 553142). The panel of fluorophore-conjugated antibodies used to identify immune cell populations residing in the uterus is listed in **Supplementary Table 2** and specific T cell subpopulations in **Supplementary Table 3**. Samples were stained for 30 minutes and fixed using either 4% PFA or the FoxP3/Transcription Factor Staining Buffer Set when detecting intracellular cytokines (Invitrogen Cat. No. 00-5523-00). 30 μL of CountBright Absolute Counting Beads (Invitrogen Cat. No. C36950) were added to the final 300 μL single cell suspension for quantification of total cell number. Compensation was performed using UltraComp eBeads (Invitrogen Cat. No. 01-2222-42). FMO controls were used during initial panel validation and again as necessary, with the inclusion of an unstimulated control during intracellular cytokine staining. Samples were processed on the BD Biosciences LSRFortessa X-20 at the Immunophenotyping Platform at the Research Institute of the McGill University Health Centre. Flow cytometry data was analyzed using the FlowJo software (BD Biosciences Version 10.8.1).

### Statistical analysis

Data shown represents mean ± SEM of independent samples. Statistical analysis comparing experimental groups was performed first by the removal of outliers (ROUT, Q=1%), and either ordinary one-way analysis of variance (ANOVA), Tukey’s multiple comparisons test or two-tailed unpaired Student t-tests as fit, using GraphPad Prism (Version 9.4.1) software. P-values less than 0.05 were considered statistically significant.

## Data availability statement

The original contributions presented in the study are included in the article/**Supplementary Material**. Further inquiries can be directed to the corresponding author.

## Ethics statement

The animal studies were approved by Animal Care Committee of the McGill University Health Centre, #MUHC 5261. The studies were conducted in accordance with the local legislation and institutional requirements. Written informed consent was obtained from the owners for the participation of their animals in this study.

## Author contributions

DD: Conceptualization, Funding acquisition, Supervision, Writing – review & editing. SY: Data curation, Formal Analysis, Investigation, Methodology, Writing – original draft. SS: Investigation, Methodology, Writing – review & editing. CP: Investigation, Writing – review & editing. PK: Investigation, Writing – review & editing. ET: Investigation, Writing – review & editing. MP: Investigation, Writing – review & editing.
