# Supplementary material for: Uterine Nodal expression supports maternal immunotolerance and establishment of the FOXP3+ regulatory T cell population during the preimplantation period
Source: Front Immunol. 2023 Oct 30;14:1276979. doi: 10.3389/fimmu.2023.1276979 (PMC10646213; doi:10.3389/fimmu.2023.1276979)
Supplement: Supplementary file 1 [file DataSheet_1.pdf]

Supplementary Material

1.1 Supplementary Figures

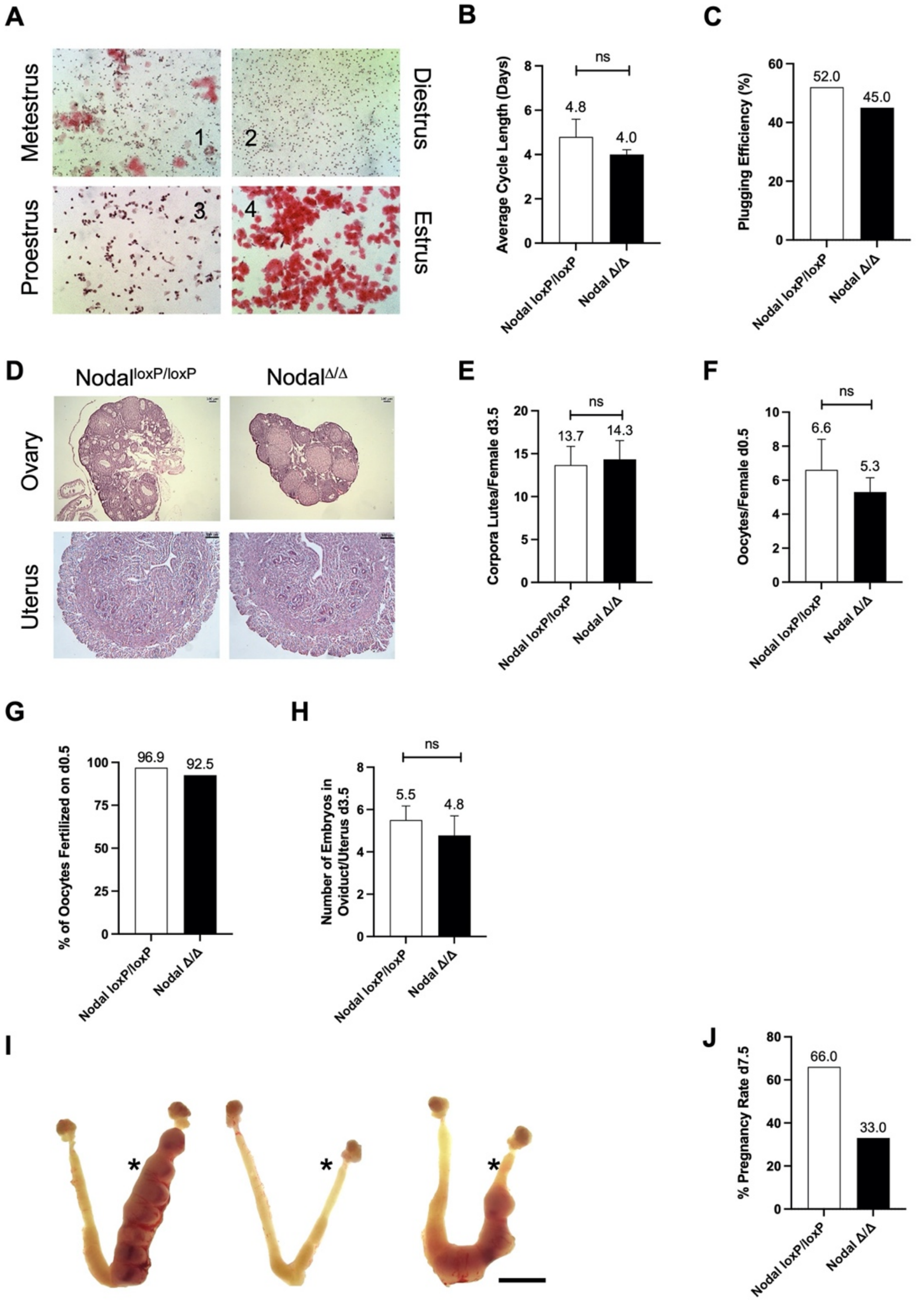

Supplemental Figure 1: Nodal$^{\Delta/\Delta}$ females cycle through estrous and have a normal reproductive tract morphology, while embryos derived from Nodal$^{\Delta/\Delta}$ are numerous and viable.

A) Representative example of each stage of the estrous cycle in the Nodal$^{\Delta/\Delta}$ mouse, depicting proestrus as mainly nucleated epithelial cells, estrus with vast quantities of cornified cells, metestrus as predominately leukocytes with cornified cells remaining and diestrus primarily leukocytes. Samples were counter-stained in Nuclear Fast Red and shown at 10X magnification. B) Average cycle length, determined by the duration between the first observance of estrus in each cycle, is unaffected in Nodal$^{\Delta/\Delta}$ females (Nodal$^{loxP/loxP}$ n=5, Nodal$^{\Delta/\Delta}$ n=15). C) Nodal$^{\Delta/\Delta}$ females exhibit normal mating behaviour as defined by plugging efficiency when mated with wild-type CD1 males overnight (Nodal$^{loxP/loxP}$ n=89, Nodal$^{\Delta/\Delta}$ n=232). D) Representative Nodal$^{loxP/loxP}$ and Nodal$^{\Delta/\Delta}$ d3.5 ovarian and uterine horn histosections. Scale bars indicate 100 µm. E) Quantification of total corpora lutea from each serial section, suggesting all plugged females experience the same rate of ovulation (Nodal$^{loxP/loxP}$ n=3, Nodal$^{\Delta/\Delta}$ n=3). F) Oocytes isolated from the oviducts of Nodal$^{\Delta/\Delta}$ mice on d0.5 were similar in number to Nodal$^{loxP/loxP}$ controls (Nodal$^{loxP/loxP}$ n=5, Nodal$^{\Delta/\Delta}$ n=10). G) The percentage of fertilized oocytes was similar to Nodal$^{loxP/loxP}$ controls (Nodal$^{loxP/loxP}$ n=33, Nodal$^{\Delta/\Delta}$ n=53). H) The number of embryos present in the oviduct or uterus on d3.5 was similar to Nodal$^{loxP/loxP}$ controls (Nodal$^{loxP/loxP}$ n=6, Nodal$^{\Delta/\Delta}$ n=9). I) Representative whole mount of d7.5 uteri from pseudopregnant Nodal$^{loxP/loxP}$ (left) or Nodal$^{\Delta/\Delta}$ (center and right). Seven mature blastocysts were transferred into one of the uterine horns (*) of each uterus prior to implantation. Note either complete implantation failure (center) or reduced number of sites (right) following blastocyst transfer to Nodal$^{\Delta/\Delta}$ uteri. Scale bars indicate 0.5 cm. J) Wildtype embryos transferred to Nodal$^{\Delta/\Delta}$ uteri show a reduced pregnancy rate (Nodal$^{loxP/loxP}$ n=21, Nodal$^{\Delta/\Delta}$ n=28). Data shows mean ± SEM.

1.2 Supplementary Tables

Supplemental Table 1: Primers used for quantitative PCR.

Gene	Forward Sequence (5' – 3')	Reverse Sequence (5' – 3')
Gapdh	AACTTTGCATTGTGGAAGG	ACACATTGGGGGTAGGAACA
Ihh	CTACAAGCAGTTCAGCCCCA	TGAGTTCAGACGGTCCTTGC
Lif	GGCAACCTCATGAACCAGAT	ACCATCCGATACAGCTCCAC
Muc1	AGTACCAAGCGTAGCCCCTA	AAGGGCATGAACAGCCTACC
Nr2f2	GCTCTGGGATCCTTTGGTCT	ATTGCAAGTTCCCAGCAGTG
Hoxa10	CGCTACGGCTGATCTCTAGG	CAGCCCCTTCAGAAAACAGT
Msx1	GCTGGAAGCTGAAGATGG	GGTGACTCTGGACCCACCTA

Supplemental Table 2: Fluorophore-conjugated antibodies used for identification of immune cell types.

Antibody	Fluorophore	Clone	Manufacturer	Cat. No.	Dilution
Viability dye	e506		Invitrogen	65-0866-14	1:75
CD45	AF700	30-F11	BioLegend	103128	1:150
CD11b	e450	M1/70	Invitrogen	48-0112-80	1:300
Ly6C	APC-Cy7	AL-21	BD Biosciences	560596	1:300
Ly6G	APC	1AB	Invitrogen	17-9668-80	1:150
MHCII	BV711	M5/114.15.2	BD Biosciences	563414	1:300
CD3	PerCP-Cy5.5	17A2	BioLegend	100217	1:100
CD19	BUV737	1D3	BD Biosciences	612782	1:150
CD4	PE	GK1.5	BD Biosciences	561829	1:150
CD8	PE-Cy7	53-6.7	Invitrogen	25-0081-81	1:300

Supplemental Table 3: Fluorophore-conjugated antibodies used for identification of T cell subpopulations.

Antibody	Fluorophore	Clone	Manufacturer	Cat. No.	Dilution
Viability dye	e506		Invitrogen	65-0866-14	1:100
CD45	AF700	30-F11	BioLegend	103128	1:150
CD3	BUV737	17A2	BD Biosciences	612803	1:150
CD4	APC-Cy7	RM4-5	BioLegend	100525	1:150
CD8a	PE-Cy7	53-6.7	Invitrogen	25-0081-81	1:300
TCR$\gamma\delta$	PerCP-e710	GL-3	Invitrogen	46-5711-80	1:150
FoxP3	FITC	FJK-16s	Invitrogen	11-5773-80	1:100
IL-17	e450	17B7	Invitrogen	48-7177-80	1:150
IL-13	PE	eBio13A	Invitrogen	12-7133-81	1:150
IFN-γ	APC	XMG1.2	BioLegend	505809	1:150
